# Hematopoietic stem cells produce intermediate lineage adipocyte progenitors that simultaneously express both myeloid and mesenchymal lineage markers in adipose tissue

**DOI:** 10.1080/21623945.2021.1957290

**Published:** 2021-08-18

**Authors:** Kathleen M. Gavin, Timothy M. Sullivan, Joanne K. Maltzahn, Jeremy T. Rahkola, Alistair S. Acosta, Wendy M. Kohrt, Susan M. Majka, Dwight J. Klemm

**Affiliations:** a Eastern Colorado Veterans Administration Geriatric Research, Education and Clinical Center (GRECC), Rocky Mountain Regional VA Medical Center, Aurora, CO, USA; b Division of Geriatric Medicine, Department of Medicine, University of Colorado Anschutz Medical Campus, Aurora, CO, USA; c Cardiovascular Pulmonary Research Laboratory, University of Colorado Anschutz Medical Campus, Aurora, CO, USA; d Division of Pulmonary Sciences and Critical Care Medicine, Department of Medicine, University of Colorado Anschutz Medical Campus, Rocky Mountain Regional VA Medical Center, Aurora, CO, USA; e Flow Cytometry Shared Resource, University of Colorado Cancer Center, University of Colorado Anschutz Medical Campus, Aurora, CO, USA; f Division of Pulmonary, Critical Care and Sleep Medicine, Department of Biomedical Research, National Jewish Health, Denver, CO, USA; g Charles C. Gates Center for Regenerative Medicine and Stem Cell Biology, University of Colorado Anschutz Medical Campus, Aurora, CO, USA

**Keywords:** Adipocyte, progenitor, haematopoietic stem cell, mesenchymal, myeloid

## Abstract

Some adipocytes are produced from bone marrow hematopoietic stem cells. *In vitro* studies previously indicated that these bone marrow-derived adipocytes (BMDAs) were generated from adipose tissue macrophage (ATM) that lose their hematopoietic markers and acquire mesenchymal markers prior to terminal adipogenic differentiation. Here we interrogated whether this hematopoietic-to-mesenchymal transition drives BMDA production *In vitro*. We generated transgenic mice in which the lysozyme gene promoter (LysM) indelibly labeled ATM with green fluorescent protein (GFP). We discovered that adipose stroma contained a population of LysM-positive myeloid cells that simultaneously expressed hematopoietic/myeloid markers (CD45 and CD11b), and mesenchymal markers (CD29, PDGFRa and Sca-1) typically found on conventional adipocyte progenitors. These cells were capable of adipogenic differentiation *In vitro* and *In vitro*, while other stromal populations deficient in PDGFRa and Sca-1 were non-adipogenic. BMDAs and conventional adipocytes expressed common fat cell markers but exhibited little or no expression of hematopoietic and mesenchymal progenitor cell markers. The data indicate that BMDAs are produced from ATM simultaneously expressing hematopoietic and mesenchymal markers rather than via a stepwise hematopoietic-to-mesenchymal transition. Because BMDA production is stimulated by high fat feeding, their production from hematopoietic progenitors may maintain adipocyte production when conventional adipocyte precursors are diminished.

## Introduction

Once believed to be a homogeneous and metabolically dormant tissue, adipose tissue is now recognized to play central roles in energy metabolism and inflammation, and exhibits substantial heterogeneity between different body locations, and even within the same depot. Most adipocytes are derived from mesenchymal stem cells via lineage-restricted progenitors that contribute to the adipocyte population of specific fat depots [1]. For example, adipocytes that reside in epididymal fat are derived from either Wilm’s Tumour 1 [2] or Pax-3-expressing [3] mesenchymal progenitors, while inguinal adipocytes arise from Prx-1-expressing precursors [2]. Likewise, brown adipocytes, noted for thermogenic lipid oxidation, are generated from Myf5-expressing skeletal muscle progenitors [4], while thermogenic beige adipocytes can arise from either smooth muscle cell progenitors [5] or via transdifferentiation from white adipocytes [6,7].

It was originally assumed that all adipocytes were solely derived from the mesenchymal lineage, but there is now substantial evidence that some adipocytes arise from a haematopoietic origin. Initial experiments demonstrated that green fluorescent protein (GFP)-expressing adipocytes were produced in the major adipose depots of wild type mice transplanted with bone marrow from donor mice ubiquitously expressing GFP [8–10]. Subsequent studies using either competitive bone marrow transplantation, or non-myeloablative lineage analysis models revealed that the bone marrow-derived adipocytes (BMDAs) were more abundant in gonadal rather than subcutaneous adipose tissue [8,9,11–13], and were generated from the haematopoietic lineage via myeloid intermediates [11–13]. Other laboratories have confirmed and extended these initial observations by demonstrating the production of both white [14–22] and beige/brown adipocytes [22] from bone marrow cells [5,14,18,19,22], HSCs [16,17,20,21] and myeloid cells [15] in mice. Moreover, our laboratory [11] and Ryden et al. [14,18,19] have also reported the production of BMDAs in human bone marrow transplant recipients.

We recently reported that adipose tissue macrophages (ATMs) cultured in 3-dimensional fibrin [9] or Matrigel [9,13] matrices undergo a ‘hematopoietic-to-mesenchymal’ conversion during which haematopoietic (CD45) and myeloid (CD11b) markers are lost and conventional mesenchymal adipocyte progenitor markers (e.g. CD29, Sca-1 and PDGFRalpha) were upregulated prior to adipogenic conversion. To determine whether this developmental transition occurs *in vivo*, we measured the expression of haematopoietic/myeloid and mesenchymal markers on adipose stromal cells indelibly labelled with a myeloid-specific gene promoter. We observed that a small percentage of adipose stromal cells arising from the myeloid lineage simultaneously expressed high levels of the haematopoietic/myeloid and mesenchymal progenitor markers and were capable of adipogenic conversion *in vitro* and *in vivo*. Subsequent experiments showed that mature BMDAs and conventional adipocytes expressed comparable levels of several common adipocyte markers, but minimal or no expression of either haematopoietic and mesenchymal progenitor markers, suggesting that those markers are lost simultaneously during adipogenic conversion. Thus, in contrast to our previous *in vitro* results, it appears that BMDA progenitors retain expression of both haematopoietic and mesenchymal progenitor markers until terminal adipogenic differentiation *in vivo*.

## Results

### BMDAs are generated from BM HSCs via the myeloid lineage

**Figure 1.**
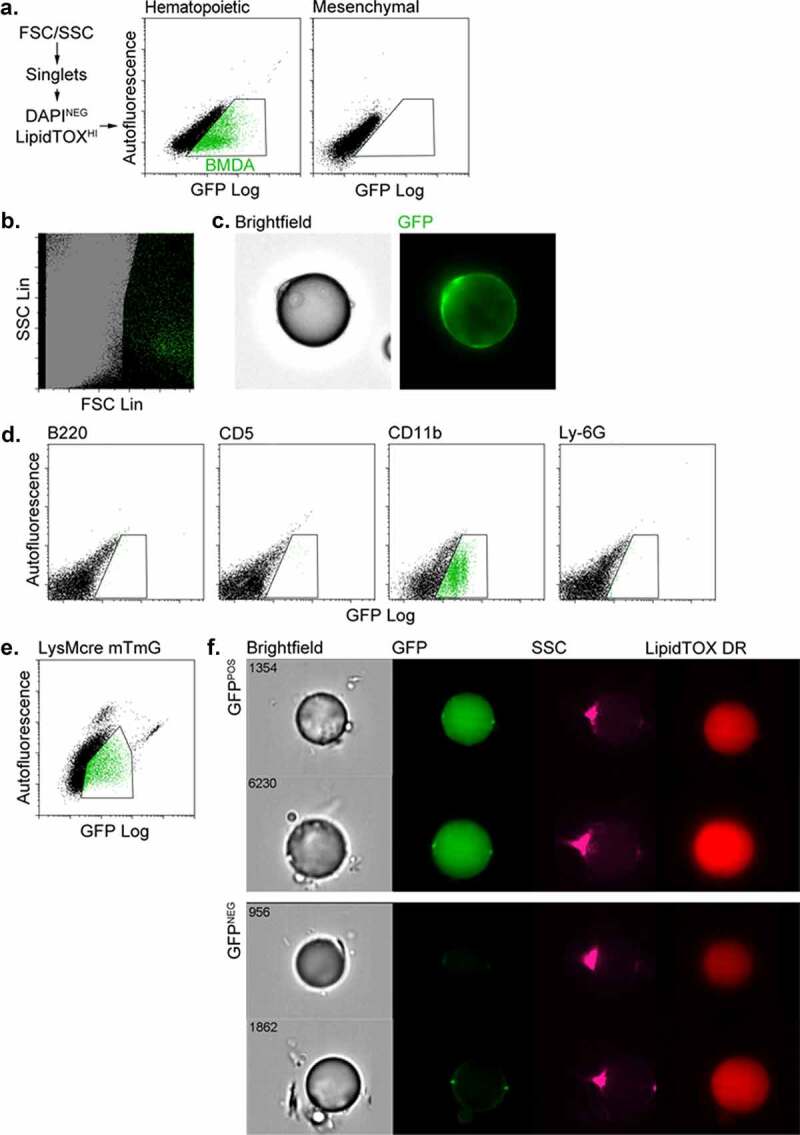
BMDAs are generated from HSCs via the myeloid lineage. (a) Competitive transplants were performed with either haematopoietic or mesenchymal progenitor cells constitutively expressing GFP, mixed with GFP-naïve carrier cells. After 8 weeks, free-floating adipocytes were prepared from adipose tissue from the transplanted mice and analysed by flow cytometry. GFP^POS^ BMDAs were detected in the adipocyte fraction of mice transplanted with GFP-labelled HSC (gated green events) but not labelled BM mesenchymal stem cells. (b) Backgating of the GFP^POS^ events in Figure 1a confirmed their large size (high forward scatter) and low internal complexity (low forward scatter) common to unilocular adipocytes. (c) Images of GFP^POS^ adipocytes from mice transplanted with GFP^POS^ HSC confirmed their identity as unilocular adipocytes with a single nucleus and cytosolic GFP fluorescence. (d) Haematopoietic sub-population cells were isolated from the BM of GFP-expressing mice with magnetic bead bearing antibodies to specific lineages (B220 = B lymphocyte, CD5 = T lymphocyte, CD11b = myeloid and Ly-6 G = granulocytes). The separated GFP^POS^ BM cells were combined with GFP^NEG^ BM cells from wild type mice to ensure survival of recipients. After 8 weeks flow cytometry showed production of BMDAs only in mice transplanted with labelled (Cd11b^POS^) myeloid cells. (e) Flow cytometry of free-floating adipocytes isolated from dual transgenic mice in which GFP expression (from an mTmG transgene) was controlled by the myeloid-specific lysozyme gene promoter (LysMcre). A population of GFP^POS^ adipocytes confirmed that some adipocytes were produced from the myeloid lineage. (f) Free-floating adipocytes were isolated from the LysMcre mTmG mice and subjected to imaging flow cytometry. Brightfield and fluorescence images confirmed the presence of GFP^POS^ unilocular (single LipidTOX stained lipid droplet) adipocytes in additional to GFP^NEG^ adipocytes

Competitive BM transplants were performed with either haematopoietic or mesenchymal progenitor cells constitutively expressing GFP, mixed with GFP naïve carrier cells. After 12 weeks, gonadal adipose tissue was isolated from the transplanted mice, digested with collagenase, and floating adipocytes were analysed by flow cytometry. GFP expressing (GFP^POS^) BMDAs were detected in the adipocyte fraction from mice transplanted with GFP-labelled BM HSC but not labelled BM mesenchymal stem cells (Figure 1a). Backgating of the GFP^POS^ events in Figure 1a confirmed their large size (high forward scatter) and low internal complexity (low side scatter) common to unilocular adipocytes (Figure 1b). Images of GFP^POS^ cells from the adipocyte fraction of adipose tissue from mice transplanted with GFP^POS^ HSC confirmed their identity as unilocular adipocytes with a single nucleus and cytosolic GFP fluorescence (Figure 1c).

Subsequent experiments employed competitive adoptive transfer to assess which haematopoietic linage(s) gave rise to BMDAs. GFP^POS^ BMDAs were only detected in mice transplanted with GFP-labelled BM myeloid cells, and no BMDAs were detected in mice transplanted with GFP^POS^ B or T lymphocytes or granulocytes (Figure 1d).

To confirm a myeloid origin for BMDAs and ensure their production was not an artefact of myeloablation, we generated mice in which myeloid cells were indelibly labelled with GFP (LysMcre-mTmG mice). GFP-expressing adipocytes were detected in these mice by flow cytometry (Figure 1e) or imaging flow cytometry (figure 1f) supporting a myeloid origin for BMDAs.

### Some ATMs possess mesenchymal progenitor cell markers and are capable of adipogenesis

We previously reported that ATMs could generate adipocytes when propagated in three-dimensional fibrin or Matrigel matrices [9,13,23]. During this conversion the cells lost expression of pan-haematopoietic (CD45) and myeloid (CD11b) markers and acquired expression of mesenchymal markers (CD29, PDGFRalpha, Sca-1) traditionally found on conventional adipocyte progenitors [1,24,25]. This led us to ask whether a similar myeloid-to-mesenchymal conversion occurs *in vivo* during the production of BMDAs.

**Figure 2.**
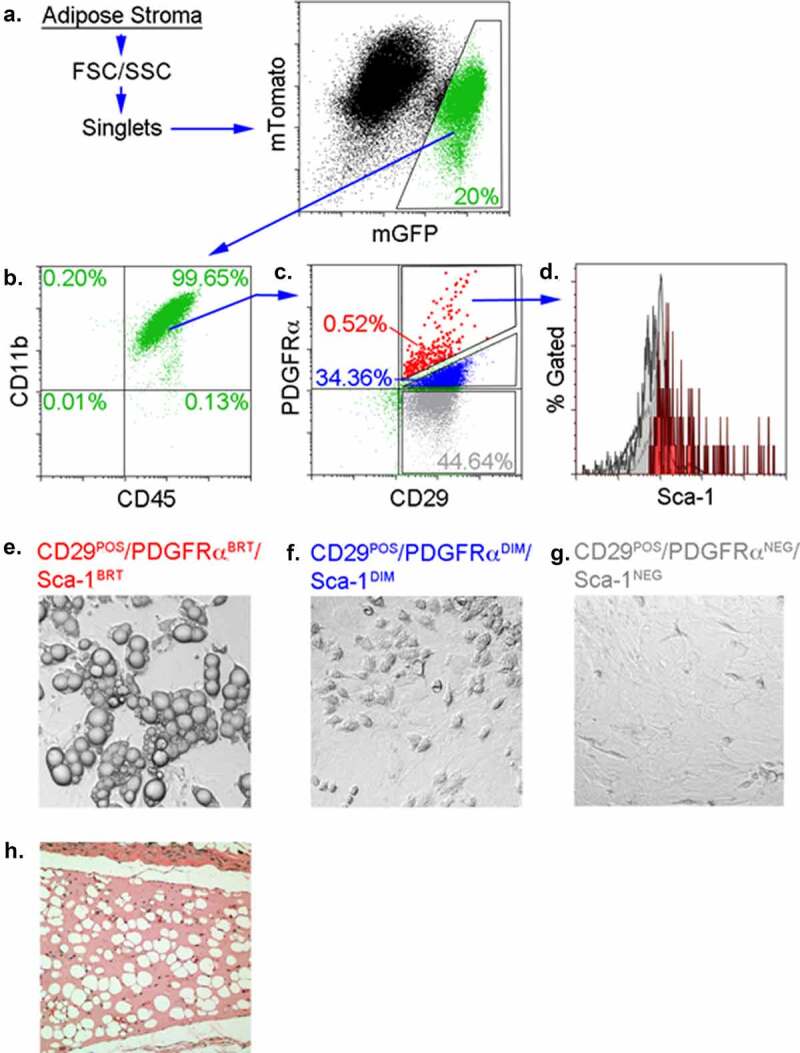
BMDAs are produced from ATM that simultaneously express haematopoietic/myeloid and mesenchymal progenitor markers. (a) Adipose tissue stromal cells from LysMcre-mTmG were separated from cell debris based on size (forward scatter, FSC) and internal complexity (side scatter, SSC), and single cells were isolated by singlet discrimination. LysM^POS^ cells were isolated based on their expression of membrane-bound GFP. (b) Approximately 99% of the LysM^POS^ cells in panel A expressed both the pan-haematopoietic marker CD45 and the myeloid marker CD11b. (c) The majority of cells in panel B also expressed the mesenchymal progenitor marker, CD29, but exhibited variable expression of PDGFRalpha (magnified red events = high expression, blue events = normal expression and grey events = no expression). Cells lacking both CD29 and PDGFR are displayed in as green events. (d) The magnified red events in panel c also expressed Sca-1, whereas other events in panel c (blue, grey and green events) were Sca-1^NEG^ (overlapping light grey peaks in panel d). (e) LysM^POS^ stromal cells expressing CD29, Sca-1 and high levels of PDGFRalpha were plastic adherent and differentiated into adipocytes when treated with MDI. The percentage of gated cells is indicated is indicated in the gates of panels b-e in font colours matching each gate. (f & g) LysM^POS^ cells expressing CD29 and only modest (blue events in panel c or no expression (grey events in panel c) of PDGFR were also plastic adherent by incapable of adipogenic conversion even when treated with adipogenic agents. (h) Spontaneous adipogenic differentiation of LysM^POS^/CD29^POS^/PDGFR^POS^/Sca-1^POS^ cells (red events in panel c) implanted subcutaneously in mice

Flow cytometry analysis of adipose stroma from LysMcre-mTmG mice showed that a substantial proportion of stromal cells expressed GFP, and thus, were derived from the myeloid lineage (Figure 2a). The majority (~99%) of these cells also expressed the pan-haematopoietic marker CD45 and the myeloid marker CD11b (Figure 2b). The majority of these myeloid cells also expressed the mesenchymal marker CD29, with high (red events), low (blue events) or no (grey events) expression of a second mesenchymal progenitor marker, PDGFR (Figure 2c). CD45^POS^/CD11b^POS^/CD29^POS^ stromal cells expressing high levels of PDGFR also expressed the progenitor marker, Sca-1 (Figure 2d), and were capable of adipogenesis in culture when treated with adipogenic agents (Figure 2e), or spontaneously when implanted subcutaneously in mice (Figure 2h). Cells expressing CD45, CD11b, and CD29 with no or low PDGFR expression were plastic adherent but did not differentiate to adipocytes even when they were maintained in culture for several weeks and their media supplemented with adipogenic agents (figure 2f, g). A small number of stromal myeloid cells lacked CD45 and CD11b, and were also negative for CD29 and PDGFRalpha expression (Figure 2c, lower left quandrant). These cells did not adhere to plastic culture surfaces and were incapable of adipogenic conversion when plated in 3-D fibrin.

### LysM^POS^ cells in BM or the circulation, with or without mesenchymal markers, are incapable of adipogenic conversion

The repertoire of specific haematopoietic and mesenchymal markers on LysM^POS^ cells was also examined on BM or circulating cells from LysMcre mTmG mice. LysM^POS^ cells from BM exhibited a similar distribution of haematopoietic and mesenchymal markers as observed in adipose tissue stroma **(Supplementary Fig. 1A)**. Most of the cells expressed CD29 with variable levels (none, low or high) of PDGFRalpha expression as observed in adipose tissue stroma. A small percentage of cells were negative for the expression of all four markers (green events). None of the BM or circulating populations were capable of adhesion to plastic, nor did they differentiate into adipocytes in 3D fibrin in vitro, or Matrigel *in vivo* (data not shown).

LysM^POS^ cells in the circulation displayed a similar distribution of CD29, Sca-1, CD45 and CD11b expression to that observed in adipose tissue stroma, although the abundance of PDGFR^HIGH^ cells was less than was measured in adipose stroma or bone marrow **(Supplementary Fig. 1B)**. Like the BM, none of the myeloid-derived cells were capable of adipogenic conversion *in vitro* or *in vivo* (data not shown).

### PDGFR-expressing HSC generate adipocytes in adipose tissue

**Figure 3.**
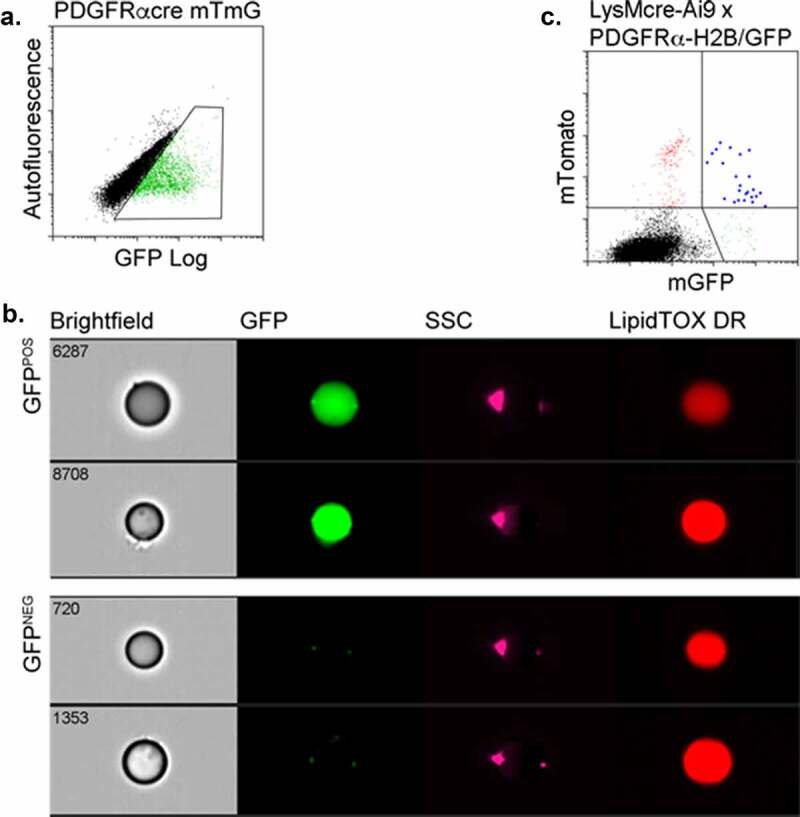
BMDAs are produced from HSCs expressing PDGFRalpha. (a & b) Wild-type mice were transplanted with HSCs from PDGFRcre-mTmG mice. After 12 weeks, adipose tissue was recovered, digested and analysed by flow cytometry. Adipocytes indelibly labelled with GFP by PDGFRalpha gene promoter activity were detected by conventional (a) or imaging (b) flow cytometry. (c) Triple transgenic donor mice were created in which mTomato expression from an Ai9 locus was controlled by cre recombinase expression regulated by the LysM gene promoter, and histone-stabilized GFP (H2B-GFP) expression was driven by the PDGFR gene promoter. HSCs from these mice were transplanted into wild-type recipients. After 8 weeks, adipocytes were isolated from the adipose tissue of the transplanted mice. Flow cytometry revealed the presence of adipocytes simultaneously expressing mTomato and H2B-GFP (blue events, upper right quadrant) indicating overlap between haematopoietic and mesenchymal developmental pathways in the production of these adipocytes

Because PDGFRalpha is a common mesenchymal marker for the adipocyte lineage and co-expressed with CD29 on putative BMDA precursors, we evaluated whether BMDAs were produced from progenitor cells expressing this marker. GFP^POS^ adipocytes were detected in the adipose tissue of mice transplanted with HSCs from donor mice in which GFP expression was controlled by the PDGFRalpha gene promoter (Figure 3a, b).

To verify the production of BMDAs from HSC via intermediates that express both myeloid and mesenchymal markers, we generated triple transgenic mice in which LysM promoter activity drove indelible mTomato expression from the Ai9 locus, and the PDGFRalpha gene promoter controlled expression of a chimeric protein composed of nuclear localized histone H2B linked to GFP. PDGFRalpha gene promoter-driven expression of H2B-GFP has previously been demonstrated in adipocyte progenitors [24,26]. In our experiments, flow cytometry of free-floating adipocytes from these mice revealed the presence of dual LysM/PDGFR-labelled adipocytes confirming their production from progenitors, sequentially or simultaneously, expressing these markers (Figure 3c). The relatively low number of the dual-labelled adipocytes likely reflects the low retention of the H2B-GFP label as PDGFRalpha gene promoter activity ceases as cells proliferate during their progression to mature adipocytes.

### BMDAs express conventional adipocyte markers but exhibit minimal expression of haematopoietic and mesenchymal progenitor markers

**Figure 4.**
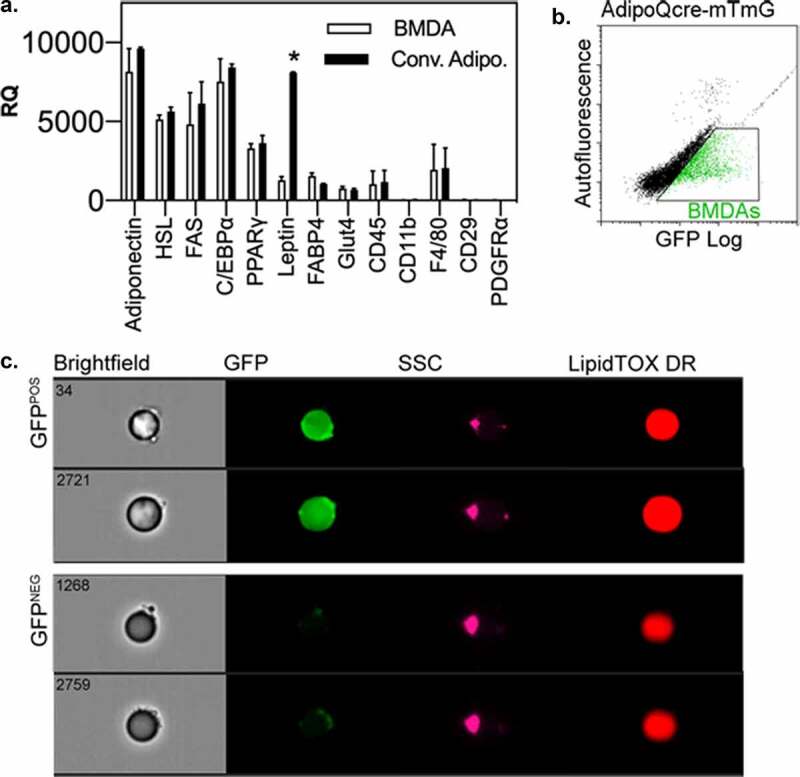
BMDAs express mature adipocytes markers, but not leptin. (a) RNA was isolated from flow cytometry purified, free floating adipocytes from wild type mice transplanted with HSCs from AdipoQcre-mTmG donors. Quantitative RT-PCR was performed with commercial primer sets. Expression of adipocyte-specific genes, with the exception of leptin, which was repressed in BMDAs, was similar between conventional and BMD adipocytes. Expression of mesenchymal and haematopoietic progenitor markers was absent or equally low in both populations. (b & c) Free floating adipocytes from wild type mice transplanted with HSCs from AdipoQcre-mTmG donors were analysed by conventional (b) and imaging (c) flow cytometry. GFP^POS^ events/adipocytes were detected among GFP^NEG^ adipocytes by both methods

BMDAs and conventional adipocytes from mouse gonadal adipose tissue were purified by flow cytometry. qRT-PCR was performed for a series of mature adipocyte markers, as well as mesenchymal and haematopoietic progenitor markers (Figure 4a). Both BMDAs and conventional adipocytes expressed common adipocyte markers including adiponectin, hormone-sensitive lipase (HSL), fatty acid synthase (FAS), C/EBPalpha and PPARgamma. However, leptin expression was significantly lower in BMDAs than conventional fat cells; a previously reported phenomenon [8,9]. The haematopoietic markers, CD45 and F4/80, were detected in both adipocyte populations at low levels, while CD11b was not detected in either population. The mesenchymal markers, CD29 and PDGFRalpha, were absent in conventional adipocytes and BMDAs. Because adiponectin expression is restricted solely to bona fide adipocytes [27], we wanted to further confirm its expression in BMDAs. Thus, we transplanted wild type mice with HSC from donors in which GFP expression was controlled by the adiponectin gene promoter (Figure 4b, c). Adiponectin^POS^ BMDAs were detected in these mice at levels comparable to that measured with other lineage labelling systems (e.g. LysMcre and PDGFRcre) used to identify BMDAs.

The repertoire of cell surface markers on buoyant BMDAs and conventional adipocytes, and the non-buoyant stromal fraction of adipose tissue were also examined by flow cytometry (Table 1). Both BMDAs and conventional adipocytes showed robust staining for CD36 (fatty acid transporter) and the lipid stain, LipidTOX, both of which were much lower or absent in adipose stromal cells. However, both BMDA and conventional adipocytes were devoid of haematopoietic and mesenchymal progenitor markers, and markers found on vascular endothelial, lymphatic endothelial, smooth muscle and neuronal cells, and a cadre of miscellaneous cell surface markers. Markers for skeletal muscle were not found in any adipose cell population. Interestingly, low-level staining for platelet/endothelial cell adhesion molecule-1 (PECAM-1) was detected on BMDAs but not conventional adipocytes. While originally detected on platelets and vascular endothelial cells, PECAM-1 is also expressed on monocytes [28] and neutrophils [29] in the vascular lumen where it may foster binding and transmigration of the these cells from the vascular lumen to the adipose mesenchyme. This may be crucial to the production of BMDAs from circulating myeloid cells.

**Table 1.** Flow cytometry markers on BMDAs, conventional adipocytes, & stromal cells in wild type mice transplanted with HSCs from UbqC-GFP donor mice

	BMDA	Conv WA	SVF
**Adipocyte**			
CD36/FAT	++	++	+/-
LipidTOX	++	++	+/-
GFP	+	-	+/-
**Hematopoietic**			
Lin	-	-	+/-
CD45	-	-	+/-
CD11b	-	-	+/-
Gr-1	-	-	+/-
B220	-	-	+/-
Thy-1	-	-	+/-
Ter119	-	-	+/-
**Progenitor**			
CD29/Itg β1	-	-	+
Sca-1	-	-	+
c-Kit	-	-	+
CD34	-	-	+
**Vascular**			
**Endothelial**			
PECAM1	+	-	+/-
VE-Cadherin	-	-	+/-
Flk-1	-	-	+/-
**Lymphatic Endothelial**			
VEGFR-3	-	-	+/-
LYVE1	-	-	+/-
**Skeletal**			
**Muscle**			
αActinin	-	-	-
Desmin	-	-	-
**Smooth Muscle**			
Itg α7	-	-	-
**Neuronal**			
NCAM	-	-	+/-
**Misc**			
PDGFRα	-	-	+/-
PDGFRβ	-	-	+/-
Notch 4	-	-	+/-
Itg alpha5	-	-	+/-

BMDA, bone marrow derived adipocyte Conv WA, conventional white adipocyte SVF, stromal vascular fraction FAT, fatty acid transporter VE, vascular endothelial Itg, integrin PDGFR, platelet-derived growth factor receptor PECAM, platelet endothelial cell adhesion molecule LYVE, lymphatic vessel endothelial receptor FLK1, fetal liver kinase 1 Sca-1, stem cell antigen 1 VEGFR-3, vascular endothelial growth factor receptor 3 Lin, lineage NCAM, neural cell adhesion molecule c-kit, stem cell factor ligand

## Discussion

While the majority of adipocytes in adipose depots arise from mesenchymal progenitor cells, results herein demonstrate that a subpopulation of adipocytes is produced from haematopoietic stem cells via circulating myeloid intermediates that co-express mesenchymal progenitor markers. This conclusion is further supported by adoptive transfer studies in which donor HSC ultimately gave rise to BMDAs indelibly labelled by either the PDGFRalpha (mesenchymal progenitor) or adiponectin (mature adipocyte) gene promoters. The three models presented here highlight the generation of BMDAs via the following pathway 1) HSCs that traffic to adipose tissue and 2) generate myeloid intermediates/adipose tissue macrophage, 3) some of which co-express mesenchymal markers. As these cells differentiate into BMDAs they express the canonical adipocyte marker, adiponectin. The LysM model was also used in non-transplant experiments confirming that BMDAs are not an artefact of myeloablation.

### BMDAs arise from HSCs via the myeloid lineage

Previous *in vitro* studies demonstrated that some adipose tissue macrophage lost haematopoietic markers and acquired mesenchymal progenitor markers while cultured in three-dimensional matrices composed of fibrin or Matrigel [9,13,23]. When removed from the 3-dimensional matrices the haematopoietic marker-depleted and mesenchymal marker-enriched cells were capable of adipogenic differentiation in culture and *in vivo*. Our goal here was to determine whether this ‘hematopoietic-to-mesenchymal’ transition also occurred *in vivo*.

We created an adoptive transfer model in which HSCs from LysMcre reporter mice were transplanted into wild type recipients. Reporter-positive cells in adipose tissue identified 1) cells with an HSC origin, and 2) cells that currently or previously expressed the LysM myeloid marker. As anticipated, we found that the vast majority of LysM^POS^ cells in adipose tissue stroma were also positive for the haematopoietic/myeloid markers, CD45 and CD11b. A substantial portion of these cells were also CD29^POS^, but only a small number of LysM^POS^ cells simultaneously expressed high levels of PDGFRalpha and Sca-1. Only LysM^POS^ stromal cells simultaneously expressing both haematopoietic/myeloid and mesenchymal markers were capable of adipogenesis via the rapid, simultaneous loss of haematopoietic and mesenchymal markers, rather than the stepwise loss of haematopoietic markers prior to acquisition of mesenchymal features.

### BMDAs are not simply lipid-engorged macrophage or foam cells

Previous flow cytometry studies and gene expression analysis of cell surface markers indicated that BMDAs, like conventional adipocytes, lack haematopoietic and mesenchymal progenitor markers [8,9]. Adipocytes generated from myeloid adipocyte progenitors in culture also lack CD11b and exhibit low expression of CD45 and F4/80, and are deficient in mesenchymal progenitor markers [23]. Here we showed that BMDAs also lack markers for other terminally differentiated lineages present in adipose tissue including vascular and lymphatic endothelial cells, skeletal and smooth muscle cells and neurons. However, they express the same markers as conventional adipocytes, including adiponectin, whose expression is restricted to bona fide adipocytes. Interestingly, leptin was reduced in BMDAs; a feature previously attributed to these cells [8,9].

BMDAs are also not lipid-engorged macrophage or foam cells. We have consistently failed to detect high-level expression of pan-leukocyte (CD45) or myeloid (CD11b, F4/80) markers on BMDAs by flow cytometry, gene microarray analysis or Q-RT-PCR [8,9,30,31]. BMDA phenotyping herein that shows no or very low expression of these markers on BMDAs amplifies this conclusion. Moreover, adiponectin gene expression, which is restricted to mature adipocytes [32], was detected in flow cytometry isolated BMDAs, whereas adiponectin expression suppresses foam cell formation [33,34]. Thus, BMDAs are not foam cells despite their myeloid origin.

### Conventional adipocytes are generated from multiple mesenchymal lineages

Kirkland, et al. reported that human visceral and subcutaneous preadipocytes exhibit different capacities for adipogenic differentiation and survival [35–37]. They also noted substantial differences in global gene expression patterns between human preadipocytes from subcutaneous, mesenteric and omental fat [37], and epididymal and perirenal preadipocytes from rats [38]. These results showed that distinct adipocyte progenitors reside in the different regional fat depots and may explain the different metabolic characteristics in adipocytes from subcutaneous adipose tissue compared to visceral adipose tissue of humans [39,40].

Ultimately, fate-mapping studies provided unambiguous evidence that different adipocyte populations have distinct developmental origins. For example, Spiegelman, et al. used *Myf5cre* and *Myh11cre*-based models to demonstrate production of brown or beige adipocytes from skeletal and smooth muscle progenitor cells, respectively [4,5]. Recent studies indicate that a number of developmental lineages specified by Wt1 [2], Prx1 [2], Pax3 [3] and Sox10 [41] also contribute to adipose tissue development and heterogeneity between depots. Although the current studies link BMDAs to myeloid progenitors expressing CD45 and CD11b, additional studies are needed to explore the relationship between BMDA progenitors and depot-restricted mesenchymal lineage markers.

### The production of adipocytes from haematopoietic lineages is supported by reports from other laboratories

Other groups have reported production of adipocytes from HSC or myeloid lineage progenitors. Guerrero-Juarez et al. [15] demonstrated the conversion of myeloid cells to fibroblasts having a dual-lineage gene expression signature in large excisional skin wounds. Additional experiments from the same study demonstrated the production of adipocytes indelibly labelled by the LacZ (myeloid), CD45 (pan-haematopoietic) and Resistin (adipocyte) gene promoters. LacZ expression was also noted in dermal papilla and sheath cells indicating that the lineage plasticity of myeloid cells extends beyond adipocytes.

This concept is supported by several reports from Ogawa and colleagues who, in addition to adipocytes [10], have demonstrated multilineage haematopoietic engraftment of fibroblast, myofibroblast and pericytes in various models [42–44]. Given the widespread contribution of HSC to multiple mesenchymal lineages these authors propose that mesenchymal progenitor cells are similar if not identical to HSC-derived fibroblasts. In support of this idea, they cited the presence of Mac1 or F4/80 expressing colonies in cultures of colony forming unit-fibroblast cells [45], and the ability of CD14^POS^ PBMC to generate multiple mesenchymal lineages *in vitro* [46]. Streiter and colleagues [47] also reported the production of adipocytes from fibrocytes, which are circulating blood cells with features of both fibroblasts and HSCs. However, our current data refutes the idea that all mesenchymal progenitors and adipocytes are produced solely from HSC-derived fibroblasts as experiments using myeloablative and non-myeloablative lineage labelling models clearly reveal population of adipocytes produced from non-myeloid (LysM^NEG^) and non-HSC precursors, respectively.

Our studies and those of other laboratories, now raise the question of why adipocytes, long considered solely a mesenchymal lineage, are produced from HSCs. Results from Guerrero-Juarez, et al. [15], support the contention that localized production of adipocytes from circulating myeloid cells in skin lesions provides for the regulated storage and release of energy during wound healing. Similarly, Xiong et al. [20,21] reported the production of adipocytes from HSCs in tumours and their contribution to tumour growth and cancer cell migration. Thus, BMDA production may support localized energy storage and release in tissues wherein the production of conventional adipocytes is restricted or absent. Our previous studies have noted an increase in BMDA production in conventional adipose depots in animals fed a high-fat diet or treated with adipogenic thiazolidinediones [8]. Interestingly, both conditions reduce the abundance of conventional adipocyte progenitors in adipose tissue [48–52]. Therefore, we postulate that the generation of BMDAs may serve to maintain new adipocyte production even when the production of conventional adipocytes is diminished.

Likewise, leptin-diminished BMDAs may have been crucial to the contribution of adipose tissue to metabolic regulation during early human evolution. Leptin participates in energy metabolism via its roles in short-term feeding behaviour and long-term energy expenditure [53]. During early human evolution, a period characterized by the restricted availability of dietary calories, the production of leptin-diminished BMDAs may have provided a solution for long-term energy storage without depletion of energy stores due to the penalty of leptin-induced energy expenditure.

### Summary

BMDAs are produced from HSCs via the myeloid lineage. In this model, some ATM acquire mesenchymal progenitor markers prior to terminal adipogenesis. Haematopoietic and mesenchymal markers appear to be lost simultaneously prior to or during adipogenic conversion, and not in a stepwise haematopoietic-to-mesenchymal transition. BMDAs express conventional adipocyte markers including adiponectin, but have very low leptin gene expression and do not express haematopoietic or mesenchymal progenitor markers.

## Materials and methods

### Animal Models

All procedures and treatments were approved by the Institutional Animal Care and Use Committee at the University of Colorado Anschutz Medical Campus. Mice were purchased from The Jackson Laboratory and included wild-type C57Bl/6 J (cat. no. 000664); C57Bl/6-Tg (UBC-GFP)30Scha/J (UbqC-GFP, cat. no. 004353); B6.129P2-Lyz2^tm1(cre)Ifo^/J (LysMcre, cat. no. 018956); C57BL/6-Tg (Pdgfra-cre)1 Clc/J, (PDGFRcre, cat. no. 013148); B6; FVB-Tg(Adipoq-cre)1Evdr/J, (AdipoQcre, cat. no. 010803); B6.129 (Cg)-Gt (ROSA)26Sor^tm4(ACTB-tdTomato,EGFP)Luo^/J) (mT/mG, cat. no. 007676); B6.129S4-*Pdgfra^tm11(EGFP)Sor^*/J (PDGFR-H2B/GFP, cat. no. 007669) and B6.Cg-*Gt(ROSA)26Sor^tm9(CAG-tdTomato)Hze^*/J (Ai9, cat.no. 007909).

### Materials

PCR Primers: Mouse genotyping was performed with the validated primers sets listed in **Supplementary Table 1**. Pre-optimized primers for qRT-PCR for the genes listed in **Supplementary Table 2** were purchased from Integrated DNA Technologies (Coralville, IA) or Qiagen (Germantown, MD) as indicated.

Flow Cytometry Antibodies and Reagents: Flow cytometry antibodies recognizing murine cell surface markers used throughout his paper, and their vendor are listed in **Supplementary Table 3**.

Miscellaneous Reagents and Equipment: Cell culture reagents, chemical compounds, genetic reagents, major equipment, software and other materials are described in **Supplementary Table 4.**

### Adoptive Transfer

Conventional BM transplant: Recipient mice were irradiated with a split dose of 600 rads using an X ray source. Immediately following irradiation, mice were injected in the retroorbital venous plexus with 5 × 10^6^ bone marrow cells from the donor mice indicated in each experiment. At 8 weeks post-transplant, mice were assessed for chimerism by flow cytometry analysis of peripheral blood. Over 95% of peripheral blood cells expressed fluorescent lineage markers in the transplanted mice. The mice were maintained in a climate-controlled room with alternating 12-hour periods of light and dark and were fed ad libitum.

Competitive adoptive transfer: Competitive BM transplants were performed with GFP-expressing BM separated into haematopoietic stem cell (Lin^NEG^/Sca-1^POS^/Kit^POS^) or mesenchymal progenitor cell (Lin^NEG^/CD45^NEG^/CD24^POS^/PDGFR^POS^) subpopulations based on the expression of specific cell surface markers by antibody-labelled magnetic bead separation. Two rounds of magnetic separation were performed to improve separation efficiency, which was greater than 90% [9]. The separated labelled BM cells were combined with marker-negative BM cells from wild type mice to ensure survival of recipients. Engraftment of the labelled cell subpopulations was greater than 90% for all transplants as determined by flow cytometry of PBMCs.

### Fractionation of Adipose Tissue and Preparation of PBMCs and bone marrow cells

Adipocyte and stromal fractions were prepared by collagenase digestion and flotation/differential centrifugation as previously described [8]. PBMCs were isolated from defibrinated blood by centrifugation over Ficol-Paque medium. Approximately 2 ml of blood were mixed with an equal volume of PBS and gently layered over 3 ml of Ficol-Paque. The tubes were centrifuged at 500 x g for 15 minutes and the supernatant carefully removed. The PBMCs layered on top of the Ficol-Paque were recovered, washed once with PBS and resuspended in PBS or culture medium as necessary.

Bone marrow cells were collected from the same animals by dislocation and removal of the hind leg. The lower leg and tissue were dissected from the femurs and the epiphyses cut off. Using a 27 gauge needle the marrow was flushed with 5 ml cold HBSS + 2% FBS into a collection tube then filtered through a 100 µm cell strainer. The cells were pelleted and washed with HBSS + 2% FBS.

Sorted myeloid cells were pellet at 300 g x 10 minutes and resuspend in MesenCult Basal medium plus Stem Cell Stimulatory Supplements and replated at 40,000 cells directly into one well of 96 well plate.

### Flow Cytometry

Staining of Adipocytes with DAPI and LipidTOX Deep Red: Buoyant adipocytes resulting from collagenase digestion and flotation/differential centrifugation were transferred to a clean tube and fixed with 4% paraformaldehyde in PBS for 20 minutes at room temperature. The fat cells were then transferred to another clean tube and fresh PBS added with gentle mixing. After the adipocytes accumulated at the top of the liquid, they were transferred to another fresh tube and washed with PBS two more times.

The cells were then incubated in a 1:200 dilution of LipidTox DR for 20 minutes at room temperature. DAPI was added to a final concentration of 3.75 ng/ml and incubation continued for another 10 minutes. The cells were washed and then examined by fluorescence microscopy or flow cytometry.

Conventional flow cytometry and sorting: Adipose stroma, BM cells, PBMC or adipocytes were stained with antibodies to the cell surface markers indicated in the figure legends. Gating strategies included exclusion of red blood cells with Ter199, and doublet discrimination. Controls included unstained cells and cell suspensions incubated with APC- or PE-conjugated isotype matched control antibodies. Cells were analysed or sorted using a Legacy Moflo cell sorter with Summit 4.3 software.

Imaging flow cytometry: Speed Beads were added to each sample of free-floating adipocytes for synchronization of the detectors and flow parameters. Data was acquired on an ImageStream X cytometer for brightfield (BF), side scatter (SSC), GFP fluorescence, and LipidTOX fluorescence using LED trans-illumination, 2 mW 785 nm and 100 mW 488 nm lasers, respectively. Gating parameters were determined post-hoc by IDEAS software with preliminary gating to separate intact cells from cell debris and synchronization beads. Images of cells with best focal quality were gated using a metric for image clarity (Gradient RMS of the BF image). Single colour controls were used to create a compensation matrix that was applied to all sample files. Following data acquisition, adipocytes were observed in the brightfield imagery. Approximately 25 events of each type were used to create ‘true’ populations and IDEAS software was used to compare the mean value for 25 gating features between the ‘true’ populations. The Rd values for each feature were ranked from highest to lowest, and the features with highest Rd were selected for further gating to distinguish the true populations.

### In Vivo Adipogenesis

Flow cytometry-purified adipose tissue stromal cells were gently resuspended in 200 ul of low growth factor Matrigel at 4°C. Female athymic mice were lightly anesthetized and the Matrigel/cell suspensions were injected subcutaneously into the abdomen anterior of the thigh. After 4 weeks the solid Matrigel plugs and adjacent skin and subcutaneous muscle were removed. The plugs were fixed overnight in 4% paraformaldehyde, and then sliced in half with a razor blade. The halves were oriented in paraffin blocks with the cut surfaces up for sectioning. Five um sections were stained with haematoxylin and eosin for histological examination.

### Microscopy

Microscopy was performed on a Nikon TE2000-U inverted epifluorescent microscope with a motorized, remote focus/Z-axis stage controller. Brightfield and fluorescent images were captured to a desktop computer with either a DS-Fi2 colour camera or a DS-QiMc black & white camera both managed by a DS-U3 PC-based camera control unit. Image data was acquired with NIS-Elements software.

## PCR

Conventional PCR: DNA was extracted from flow-sorted adipocytes using DNeasy Blood and Tissue Kit reagents and columns. Genomic DNA was amplified with RED Extract-n-AMP reagent in reactions containing 500 nM of each of the appropriate forward and reverse primers. A touch-down protocol was used in which the annealing temperature was lowered from 70°C to 60°C in 0.5°C increments per cycle. Annealing was carried out for 45 seconds and the other parameters included denaturation at 94°C for 1 minute and elongation at 72°C for 2 minutes. Following the series of touchdown cycles, an additional 32 cycles were performed with the 60°C annealing conditions. PCR was performed with denaturation at 94°C for 1 minute, annealing at 63°C for 45 seconds and elongation at 72°C for 2 minutes for 31 cycles.

## Supplementary Material

Supplemental Material
