# Supplementary material for: Hematopoietic stem cells produce intermediate lineage adipocyte progenitors that simultaneously express both myeloid and mesenchymal lineage markers in adipose tissue
Source: Adipocyte. 2021 Aug 18;10(1):394–407. doi: 10.1080/21623945.2021.1957290 (PMC8381847; doi:10.1080/21623945.2021.1957290)
Supplement: Supplemental Material [file KADI_A_1957290_SM5535.zip › Adipocyte_Klemm_Supplementary.docx]

**SUPPLEMENTARY TABLES AND SUPPLEMENTARY FIGURE 1**

**Supplementary Table 1:** List of conventional PCR primers and sequences.

| **Primer Name** | **Sequence (3’ -> 5’)** | **Description** |
| --- | --- | --- |
| oIMR9020 | AAG GGA GCT GCA GTG GAG TA | Ai9 genotyping (reaction A WT F) |
| oIMR9021 | CCG AAA ATC TGT GGG AAG TC | Ai9 genotyping (reaction A WT R) |
| oIMR9103 | GGC ATT AAA GCA GCG TAT CG | Ai9 genotyping (reaction A MUT R) |
| oIMR9105 | CTG TTC CTG TAC GGC ATG G | Ai9 genotyping (reaction A MUT F) |
| oIMR7318 | CTC TGC TGC CTC CTG GCT TCT | mT/mG genotyping (common) |
| oIMR7319 | CGA GGC GGA TCA CAA GCA ATA | mT/mG genotyping (WT R) |
| oIMR7320 | TCA ATG GGC GGG GGT CGT T | mT/mG genotyping (WT F) |
| oIMR3068 | TTA CAG TCG GCC AGG CTG AC | LysM-cre genotyping (WT) |
| oIMR3067 | CTT GGG CTG CCA GAA TTT CTC | LysM-cre genotyping (COM) |
| oIMR3066 | CCC AGA AAT GCC AGA TTA CG | LysM-cre genotyping (MUT) |
| oIMR1084 | GCG GTC TGG CAG TAA AAA CTA TC | cre transgene forward |
| oIMR1085 | GTG AAA CAG CAT TGC TGT CAC TT | cre transgene reverse |
| oIMR7338 | CTA GGC CAC AGA ATT GAA AGATCT | internal positive control forward |
| oIMR7339 | GTA GGT GGA AAT TCT AGC ATC ATC C | internal positive control reverse |
| oIMR0872 | AAG TTC ATC TGC ACC ACC G | UBC-eGFP genotyping |
| oIMR1416 | TCC TTG AAG AAG ATG TGT CG | UBC-eGFP genotyping |
| oIMR7801 | CCC TTG TGG TCA TGC CAA AC | Pdgfra-H2B-eGFP genotyping (WT F) |
| oIMR7802 | GCT TTT GCC TCC ATT ACA CTG G | Pdgfra-H2B-eGFP genotyping (WT R) |
| oIMR7919 | ACG AAG TTA TTA GGT CCC TCG AC | Pdgfra-H2B-eGFP genotyping (MUT R) |

**Supplementary Table 2:** List of quantitative real-time PCR assays.

| **Gene** | **Vendor** | **Assay Identifier** |
| --- | --- | --- |
| *Ccl2* | Integrated DNA Technologies | Mm.PT.58.42151692 |
| *Tnf* | Integrated DNA Technologies | Mm.PT.58.12575861 |
| *Tbp* | Integrated DNA Technologies | Mm.PT.39a.22214839 |
| *Il6* | Integrated DNA Technologies | Mm.PT.58.10005566 |
| *Ccl3* | Integrated DNA Technologies | Mm.PT.58.29283216 |
| *B2m* | Integrated DNA Technologies | Mm.PT.39a.22214835 |
| *Lep* | Integrated DNA Technologies | Mm.PT.58.13515402 |
| *Adipoq* | Integrated DNA Technologies | Mm.PT.58.9719546 |
| *Ccl7* | Integrated DNA Technologies | Mm.PT.58.17719534 |
| *eGFP* | Qiagen | QT01171611 |
| *Ucp1* | Qiagen | QT00097300 |
| *Pdgfra* | Qiagen | QT00140021 |
| *Actb* | Qiagen | QT01136772 |
| *Plin1* | Qiagen | QT00150360 |
| *Itgb1* | Qiagen | QT00155855 |
| *Gapdh* | Qiagen | QT01658692 |
| *Pdk4* | Qiagen | QT00157248 |

**Supplementary Table 3:** Flow cytometry antibodies and fluorophore conjugates

| **Antibody** | **Fluorophore** | **Vendor** | **Catalog** |
| --- | --- | --- | --- |
| CD11b | PE/Cy7 | BioLegend | cat no. 101215 |
| CD45 | APC/Cy7 | BioLegend | cat no. 103115 |
| CD29 | PE/Cy5 | BioLegend | cat no. 102219 |
| CD140a | APC | BioLegend | cat no. 135907 |
| Sca-1 | AF700 | BioLegend | cat no. 108141 |
| B220 | PE/Cy5 | BioLegend | cat no. 103209 |
| CD5 |  |  |  |
| Ly6G/Ly6C | PE/Cy5 | BioLegend | cat no. 108409 |
| Sca-1 | BV650 | BioLegend | cat no. 108143 |
| CD170(Siglec-F) | APC | BioLegend | cat no. 155507 |
| CD11b | PE/Cy7 | BioLegend | cat no. 101215 |
| mouse FcX | - | BioLegend | cat no. 101319 |
| Compensation beads | - | eBiosciences | cat no. 01-1111-42 |

**Supplementary Table 4:** List of key resources and reagents.

| **Reagent Type** | **Description** | **Vendor** | **Identifiers** |
| --- | --- | --- | --- |
| Antibody-linked magnetic beads | CD45 MicroBeads, mouse | Miltenyi Biotech | Cat. No. 130-052-301 |
| Antibody-linked magnetic beads | CD5 (Ly-1) MicroBeads, mouse | Miltenyi Biotech | Cat. No. 130-049-301 |
| Antibody-linked magnetic beads | CD11b MicroBeads, human & mouse | Miltenyi Biotech | Cat. No. 130-049-601 |
| Antibody-linked magnetic beads | Anti-Ly-6G, MicroBeads, mouse, Ultrapure | Miltenyi Biotech | Cat. No 130-120-357 |
| Antibody-linked magnetic beads | CD45B (B220) MicroBeads, mouse | Miltenyi Biotech | Cat. No. 130-049-501 |
| Antibody-linked magnetic beads | Lineage Cell Depletion Kit, mouse | Miltenyi Biotech | Cat. No. 130-090-858 |
| Biological molecule | Fibrinogen | Sigma Aldrich | cat no. F8630 |
| Biological molecule | Thrombin | Sigma Aldrich | cat no. T9549 |
| Biological molecule | Bovine plasminogen | Enzyme Research Laboratories | cat no. BPg |
| Biological molecule | Urokinase | Sigma Aldrich | cat no. U4010 |
| Biological molecule | Matrigel | Corning | Cat. No. [354234](https://ecatalog.corning.com/life-sciences/b2c/US/en/Surfaces/Extracellular-Matrices-ECMs/Corning%C2%AE-Matrigel%C2%AE-Matrix/p/354234) |
| Cell culture reagent | Dulbeco's modified eagle medium | MediaTech | cat no. 10-013-CV |
| Cell culture reagent | Fetal bovine serum |  |  |
| Cell culture reagent | MesenCult Basal mouse media | StemCell Technologies | cat no. 05501 |
| Cell culture reagent | Stem Cell Stimulatory Supplement | StemCell Technologies | cat no. 05502 |
| Cell Separation Column | LD Columns | Miltenyi Biotech | 130-042-901 |
| Chemical compound | DAPI | ThermoFisher Scientific | cat no. D1306 |
| Chemical compound | Paraformaldehyde | Electron Microscopy Sciences | cat. RT 15710-S |
| Chemical compound | HCS LipidTOX Deep Red Neutral Lipid Stain | ThermoFisher Scientific | cat no. H34477 |
| Commercial kit | Dneasy Blood and Tissue kit | Qiagen |  |
| Commercial kit | RED Extract-n-amp | Sigma Aldrich | cat no. R4775 |
| Genetic reagent sample (*M. musculus*) | C57BL/6J | Jackson Laboratory | stock no. 000664 |
| Genetic reagent sample (*M. musculus*) | LyzM-cre | Jackson Laboratory | stock no. 004781 |
| Genetic reagent sample (*M. musculus*) | mT/mG | Jackson Laboratory | stock no. 007676 |
| Genetic reagent sample (*M. musculus*) | Pdgfra-cre | Jackson Laboratory | stock no. 013148 |
| Genetic reagent sample (*M. musculus*) | AdipoQ-cre | Jackson Laboratory | stock no. 010803 |
| Genetic reagent sample (*M. musculus*) | Pdgfra-H2B-eGFP | Jackson Laboratory | stock no. 007669 |
| Genetic reagent sample (*M. musculus*) | Ai9 | Jackson Laboratory | stock no. 007909 |
| Instrument | Imaging Cytometer | Amnis | ImageStream X MkII |
| Instrument | Flow Sorter | Beckman Coulter | MoFlo XDP70 |
| Instrument | Fluorescent Microscope | Nikon | TE2000-U inverted epifluorescent microscope |
| Instrument | Color Camera &  Black & White Camera  Camera Control Unit | Nikon | DS-Fi2 color camera &  DS-QiMc black white camera &  DS-U3 camera control unit |
| Software | Summit 4.3 | Beckman Coulter |  |
| Software | IDEAS | Amnis |  |
| Software | Prism 9.1 | GraphPad |  |
| Software | NIS Elements Software | Nikon |  |


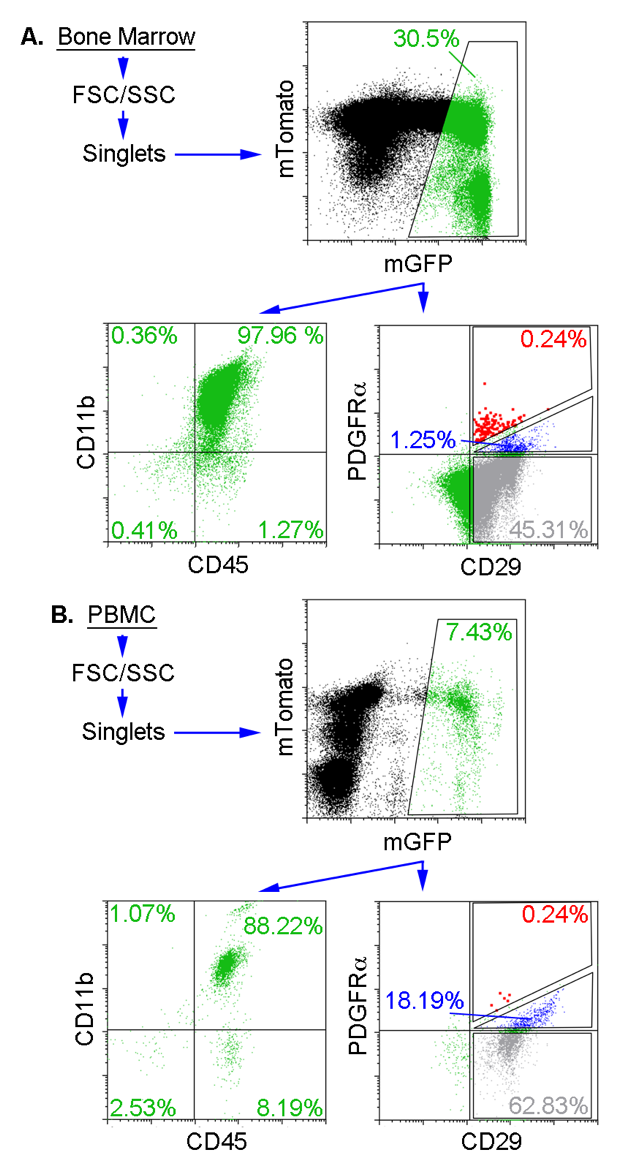


**Supplementary Figure 1. Myeloid cells that simultaneously express hematopoietic/myeloid and mesenchymal progenitor markers are also present in BM and the circulation, but do generate BMDAs. A)** BM and **B)** peripheral blood mononuclear cells from LysMcre-mTmG mice were assessed for the presence of dual lineage BMDA progenitors using the flow cytometry sorting scheme described for Figure 2. Live cells were separated from cell debris based on size (forward scatter, FSC) and internal complexity (side scatter, SSC), and single cells were isolated by singlet discrimination. LysM^POS^ cells were isolated based on their expression of membrane-bound GFP. The majority of the LysM^POS^ cells in expressed both the pan-hematopoietic marker CD45 and the myeloid marker CD11b. The majority of cells also expressed the mesenchymal progenitor marker, CD29, but exhibited variable expression of PDGFRα (red events = high expression, blue events = normal expression and grey events = no expression). Cells lacking both CD29 and PDGFRα are displayed as green events. None of the cell populations recovered from BM or PBMC were capable of adipogenic conversion on plastic before or after passage through Matrigel, or when implanted in vivo.
